# Advanced therapies for inherited optic neuropathies

**DOI:** 10.1038/s41433-025-04109-1

**Published:** 2025-11-29

**Authors:** David Chuen Soong Wong, Rahul Makam, Patrick Yu-Wai-Man

**Affiliations:** 1 John van Geest Centre for Brain Repair and MRC Mitochondrial Biology Unit, Department of Clinical Neurosciences, University of Cambridge, Cambridge, UK; 2 Cambridge Eye Unit, Addenbrooke’s Hospital, Cambridge University Hospitals NHS Foundation Trust, Cambridge, UK; 3 Moorfields Eye Hospital NHS Foundation Trust, London, UK; 4 Institute of Ophthalmology, University College London, London, UK

**Keywords:** Visual system, Disease genetics, Hereditary eye disease, Optic nerve diseases

## Abstract

Inherited optic neuropathies (IONs), such as Leber hereditary optic neuropathy (LHON) and autosomal dominant optic atrophy (ADOA), typically lead to irreversible severe vision loss due to mitochondrial dysfunction causing retinal ganglion cell degeneration. Although current treatment options are limited, substantial progress has been made recently in our understanding of the molecular genetic pathways that lead to retinal ganglion cell loss. Clinical trials for LHON have demonstrated the efficacy of idebenone, an oral neuroprotective agent, and gene replacement therapy using allotopic gene expression. Early phase clinical trials are underway for ADOA caused by variants in the nuclear gene *OPA1* using innovative techniques to modulate gene expression in a variant-agnostic manner. In this review, we have critically appraised a range of therapeutic strategies, including gene editing and stem cell-based optic nerve regeneration, with a discussion of the barriers to translation. Future studies focussing on understanding genetic heterogeneity, disease variability and optimising patient selection for clinical trials are essential to improve patient management and fast track transformative therapies for IONs.

## Introduction

Inherited optic neuropathies (IONs) are among the leading causes of blindness in children and young adults [1, 2]. The prevalence of IONs is around 1 in 25,000 in the United Kingdom [3, 4], with a significant impact on quality of life and high societal costs due visual loss occurring during the most productive years of life [3, 5–7]. The commonest IONs are Leber hereditary optic neuropathy (LHON, OMIM 535000) and autosomal dominant optic atrophy (ADOA, OMIM 165500), which share common pathophysiological mechanisms with mitochondrial dysfunction precipitating retinal ganglion cell (RGC) loss, but they differ in their clinical presentation and genetic causes.

LHON typically presents in young men with bilateral, painless, central vision loss, either simultaneous at onset or sequential with the fellow eye being affected within a few weeks [8–11]. Vision rapidly deteriorates over weeks to months, at which point a nadir is typically reached, after which visual function usually plateaus. Some patients can experience a partial, spontaneous recovery of vision, but this is dependent on the causative mitochondrial DNA (mtDNA) variant and age of onset [11]. In contrast, ADOA has a more gradual onset, usually presenting in childhood with insidious, progressive vision loss and dyschromatopsia, involving both eyes symmetrically [12]. Despite the differences in clinical progression, both diseases are characterised by preferential RGC degeneration secondary to mitochondrial dysfunction [13, 14].

Three mtDNA variants account for ~90% of LHON cases: m.3460 G > A in *MT-ND1*, m.11778 G > A in the *MT-ND4* gene, and m.14484 T > C in *MT-ND6* [4]. All three genes encode key subunits of complex I of the mitochondrial respiratory chain. The remaining LHON cases are caused by rarer pathogenic mtDNA variants or recessive variants in nuclear genes, such as *DNAJC30*, *NDUFS2* and *NDUFA12* [14]. In contrast, over 60% of ADOA are caused by pathogenic variants in the nuclear gene *OPA1*. The OPA1 protein plays a critical role in mitochondrial fusion, the stability of the mitochondrial respiratory chain, calcium homeostasis and the regulation of apoptosis [15]. Over 500 disease-causing variants in the *OPA1* gene have been identified [16, 17], and there is an expanding list of other causative nuclear genes, highlighting the complex genetic basis of ADOA [14, 15]. Incomplete penetrance is observed in ION carriers [18], which complicates diagnosis, prognostication and genetic counselling.

Current treatments for IONs are limited and there is a pressing need for therapies that protect and restore visual function. Emerging approaches include pharmacological protection of mitochondrial function, gene therapy to correct or replace defective variants, gene expression modulation, and stem cell-based strategies for optic nerve regeneration. However, challenges such as the relatively low disease prevalence, genetic heterogeneity, and incomplete penetrance complicate the clinical translation of these therapies. Nevertheless, great progress has been made in recent years, particularly in neuroprotection and gene therapy, offering hope for patients who previously faced irreversible visual loss.

This review will explore the current landscape of therapeutic strategies for IONs, evaluating their development, clinical potential, and the challenges of proving their efficacy to the regulators.

## Pharmacological neuroprotection

The degeneration of RGCs in IONs is driven by mitochondrial dysfunction. Several agents are being investigated for their potential to stabilise mitochondrial function and reduce oxidative stress to preserve vision.

**Fig. 1:**
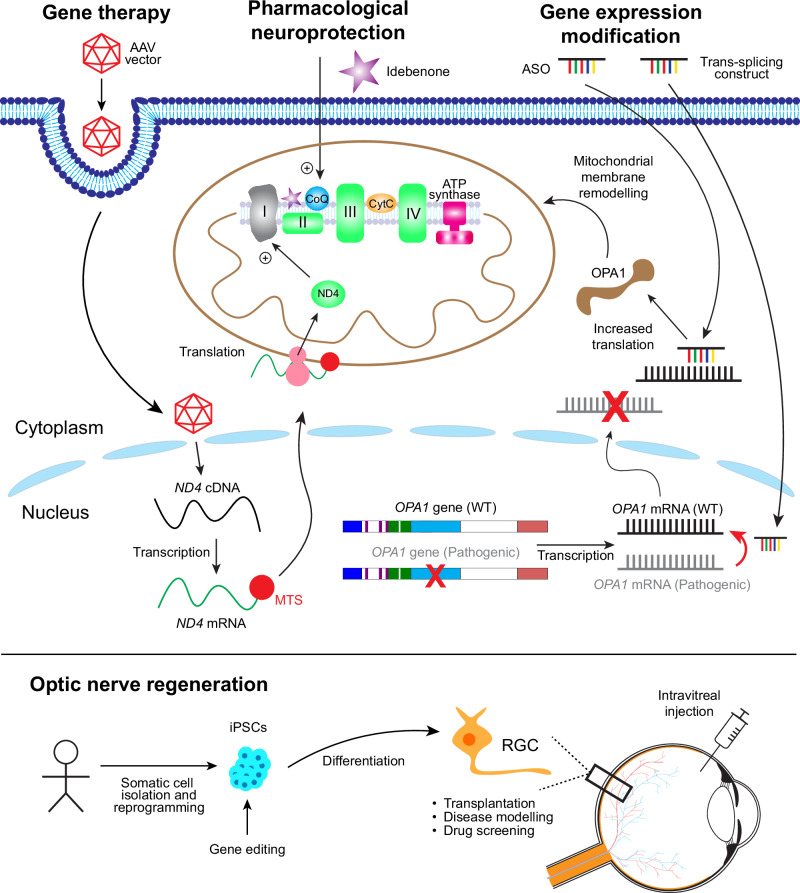
Therapeutic strategies for IONs. Mitochondrial dysfunction leading to RGC degeneration is the hallmark of IONs. In LHON, pathogenic variants in subunits of Complex I (grey) lead to a defective electron transfer along the mitochondrial respiratory chain complexes. **Pharmacological neuroprotection** with oral idebenone, which is a ubiquinone, partially restores mitochondrial function by bypassing Complex I. In contrast, **gene therapy** vectors are delivered via intravitreal injection to reach RGCs. For LHON patients carrying the m.11778 G > A variant in *MT-ND4*, the functional wild-type (WT) ND4 protein is reintroduced via allotopic gene expression using a construct that includes a mitochondrial targeting sequence (MTS). In most cases of ADOA, a loss of function variant in the nuclear *OPA1* gene results in insufficient OPA1 protein being generated (haploinsufficiency) to sustain normal mitochondrial physiology. **Gene expression modification** with ASOs or trans-splicing acts at the level of the mRNA, boosting translation to generate more wild-type OPA1 protein to rescue mitochondrial function or correcting pre-mRNAs with pathogenic variants, respectively. Finally, preclinical studies are ongoing to optimise **optic nerve regeneration** strategies, for example with RGCs derived from iPSCs.

One of the most extensively studied neuroprotective agents is idebenone, a synthetic analogue of coenzyme Q10. It primarily functions to bypass a defective complex I in the electron transport chain, thereby enhancing mitochondrial respiration (Fig. 1) [19, 20]. An early randomised, double-blind, placebo controlled trial (RHODOS), alongside an expanded access programme, natural history case record survey, and several cohort studies and case reports, demonstrated that idebenone is safe and more effective in improving visual acuity when initiated early in the disease course [21–23]. While RHODOS failed to demonstrate a statistically significant difference in best BCVA at endpoint, a numerical advantage of idebenone was shown (+6 ETDRS letters in treated eyes, compared with +3 in placebo eyes), which alongside the consistent trend of benefit in other datasets led to marketing approval under exceptional circumstances by the European Medicines Agency (EMA) for use in LHON in 2015 [24]. This decision was guided by the rarity of LHON, lack of treatments, and already commonplace off-label use of idebenone, complicating the procurement of more comprehensive placebo-controlled data.

One of the conditions of EMA approval was the conduction of a natural-history controlled, open-label interventional study. The LEROS study subsequently assessed the safety and efficacy of idebenone in 199 patients with LHON within five years of symptom onset across multiple centres internationally [25]. Over 24 months, patients received idebenone at 900 mg/day and were compared with a historical control group. The primary endpoint was the rate of clinically relevant benefit (CRB) at 12 months, defined as either a clinically relevant recovery (CRR, an improvement from an off-chart acuity to reading at least 5 ETDRS letters, or a + 10 letter improvement if already on-chart) or a clinically relevant stabilisation (CRS, a maintenance of >35 ETDRS letters) [25]. At 12 months, 42.3% of idebenone-treated patients in the acute phase (up to one year since onset of vision loss) achieved CRB compared to 20.7% in the control group (*p* = 0.002). This effect was maintained at 24 months (52.9% in the treated group vs. 36.0% in the control group, *p* = 0.03). There was also significantly higher frequency of CRB in treated chronic eyes (50.3%) compared with controls (38.6% *p* = 0.009), which was sustained at 24 months. The degree of visual improvement varied based on factors such as the disease phase, mtDNA variant, age, and sex, with the m.11778 G > A variant showing the greatest response to treatment. Eyes with the m.3460 G > A variant did not benefit. Idebenone was well-tolerated with a favourable safety profile similar to previous studies [21, 23]. The LEROS study therefore confirms the utility of idebenone as a therapeutic option for patients with LHON in both the acute and chronic phases. While limited by the lack of a placebo arm, the use of natural history controls had been previously agreed by the EMA as a feasible approach for the investigation of idebenone efficacy, in recognition of disease rarity and widespread clinical use of idebenone [24].

Given the shared mechanisms of RGC degeneration between LHON and ADOA, idebenone has been trialled off-label for ADOA. A pilot study demonstrated some visual function recovery in five of seven ADOA patients after at least one year of idebenone treatment [26]. A subsequent larger retrospective case-control study from the same group of investigators reported significant improvement in visual acuity in treated patients compared with untreated controls [27]. However, this evidence is limited, and an adequately powered, randomised, placebo-controlled trial is needed to better evaluate the efficacy of idebenone in ADOA.

Other neuroprotective agents that have been investigated include elamipretide, a small peptide that reduces mitochondrial reactive oxygen species production. In a randomised controlled trial for LHON patients with chronic vision loss, elamipretide eye drops did not meet the primary endpoint, but showed some benefits in a two-year open-label extension study [28]. In a small open-label trial, four of five treated LHON patients recovered vision over one year after starting oral treatment in the subacute phase with EPI-743, which is thought to have antioxidant properties [29].

Promoting mitochondrial biogenesis and optimising the clearance of dysfunctional mitochondria are other therapeutic approaches that are being explored for LHON. The pharmacological inhibition of mTOR has been shown to promote degradation of dysfunctional mitochondria and restore some mitochondrial function in heteroplasmic disease models [30, 31]. Activation of oestrogen signalling increases mitochondrial biogenesis and reduces oxidative stress, thereby rescuing mitochondrial function in a LHON in vitro model [32]. Finally, some microRNAs regulate mitochondrial biogenesis and their deactivation has been found to improve visual function in LHON mouse models [33]. These preclinical studies will need to be further validated before contemplating early phase clinical trials.

## Gene therapy

In contrast to pharmacological neuroprotection, gene therapy strategies aim to permanently modify the disease-causing genes themselves. This is a particularly appealing approach in monogenic diseases like LHON and ADOA. Techniques under investigation include allotopic gene expression, gene editing, and inducing mtDNA heteroplasmic shift.

### Allotopic gene expression in LHON

Gene therapy for primary mtDNA disease is challenging due to the physical barriers in delivering therapeutic genes or proteins into mitochondria, which have a double membrane structure. Allotopic gene expression circumvents this by engineering a re-coded version of the gene of interest with a mitochondrial targeting sequence (MTS) and delivering it to the nucleus using an adeno-associated virus (AAV) vector. The gene in the form of cDNA is transcribed to mRNA in the nucleus, which is then guided by the MTS to ribosomes on the surface of the mitochondria. Here, the mRNA is translated into protein and the nascent polypeptide chain is imported into mitochondria (Fig. 1). This is more efficient than direct import of hydrophobic proteins [34]. In preclinical studies, an AAV-delivered synthetic *ND4* gene with an MTS could rescue oxidative phosphorylation defects in vitro in cells with the m.11778 G > A LHON variant [35]. Subsequent studies with animal models showed robust expression of human ND4 protein with several different AAV vectors, associated with functional preservation of vision [36–39]. These encouraging results led to the initiation of human clinical trials.

An AAV vector was designed to deliver the replacement *MT-ND4* gene (rAAV2/2-*ND4*). Initial safety studies showed it was well-tolerated with mild uveitis being the most common adverse event [40, 41]. Phase III trials tested the efficacy and safety of rAAV2/2-*ND4* in 38 LHON-affected subjects in the subacute stage (visual loss <6 months, RESCUE trial, NCT02652767) [42], and separately in 37 subjects in the dynamic stage (visual loss of 6-12 months, REVERSE trial, NCT02652780) [43]. Both were multicentre, randomised, double-masked, sham-controlled trials. Subjects from the RESCUE and REVERSE trials were also recruited to a long-term follow up study (RESTORE trial, NCT03406104) [44]. Curiously, an unexpected bilateral gain in best-corrected visual acuity (BCVA) following unilateral drug injection was noted in these trials. After five years, these gains were sustained (+20 letters in both treated and sham eyes), associated with a meaningful increase in quality of life, and there were no serious adverse effects [44–46]. In the REFLECT trial (NCT03293524), bilateral injections of rAAV2/2-*ND4* were found to be safe and there was a trend towards a better visual outcome compared with unilateral treatment (+12 ETDRS letters compared to +8 ETDRS), in keeping with a biological dose effect [47]. Experiments with non-human primates injected in one eye with rAAV2/2-*ND4* showed the presence of the viral vector in the contralateral noninjected retina and optic nerve, as well as the optic chiasm. Although the transfer mechanism remains unclear, these findings provide a possible explanation for the bilateral improvement observed in human clinical trials. Further studies are ongoing on postmortem ocular tissues collected from two subjects who received a unilateral injection of rAAV2/2-*ND4* and died from causes unrelated to their treatment.

Bilateral visual improvement following unilateral injection prevented the intended use of untreated eyes as controls. A pooled analysis of data from the RESCUE, REVERSE, RESTORE and REFLECT trials therefore compared the overall effect of treatment with 208 matched natural history control patients [48]. There was a consistent statistically and clinically significant improvement in BCVA of +21.5 ETDRS letters in treated eyes compared with controls, with a larger treatment effect with bilateral treatment compared with unilateral treatment. Furthermore, a meta-analysis compared the data from all the rAAV2/2-*ND4* trials with published data from the literature on idebenone treatment and the natural history of LHON [49]. An intravitreal injection of rAAV2/2-*ND4* was more effective than idebenone and both were superior to natural history. It must be stressed that rAAV2/2-*ND4* remains an experimental therapy that is still awaiting approval by the EMA and the US Food and Drug Administration (FDA), and that these trials are limited by their absence of true controls. While matched natural history controls are informative, these datasets are largely retrospective and derived from clinical records. Visual acuity may improve over time in LHON as patients adapt to their residual vision, and a clinical trial setting may capture better performance in an acuity task. Prospective natural history data for LHON would help to allay this concern. Additionally, the mean BCVA for pooled, treated eyes at last observation was 1.38 logMAR; while this represents a − 0.301 logMAR improvement over natural history eyes, this is still manifestly poor acuity, and well below the standards of vision for driving in the UK. Regardless, modest improvements in vision can be clinically meaningful, particularly in a disease that is otherwise untreatable and severe.

Other gene therapy clinical trials for LHON caused by the m.11778 G > A mtDNA variant have explored different gene therapy vectors based on allotopic gene expression. A phase I trial conducted in the United States of America assessed unilateral intravitreal injection of a novel AAV vector in subjects with chronic or acute bilateral vision loss. This demonstrated safety, but limited efficacy [50–52]. In a trial from China, the rAAV2-*ND4* vector was found to be safe in nine subjects [53, 54], and better visual outcomes were associated with age, earlier treatment and baseline BCVA [55, 56]. An early phase study has been initiated to look at the safety and benefit of allotopic gene expression in subjects with LHON carrying the m.3460 G > A variant in *MT-ND1* (NCT05820152).

### Challenges in gene therapy for ADOA

There are several challenges to the development of gene therapies for ADOA. Supraphysiological levels of OPA1 protein are thought to be toxic [57], so expression levels in gene replacement therapy need to be controlled carefully. Moreover, increasing expression of wild-type OPA1 may not be effective for missense variants, which could have dominant negative effects [58–60]. It is also unclear which of the eight human isoforms of *OPA1* would be best to target since they carry out different molecular functions [61]. Moreover, the size limitation of the AAV vector has so far restricted its cargo to a single *OPA1* isoform. Finally, around 20% of patients with ADOA have multi-system involvement, including hearing loss, peripheral neuropathy, myopathy, spastic paraplegia, and chronic progressive external ophthalmoplegia [62]. Ideally, gene therapy will be able to address both ocular and systemic manifestations of ADOA.

A study using an in vivo mouse model of *OPA1*-associated ADOA tested the efficacy of intravitreal injections of an AAV vector containing a human *OPA1* cDNA that encodes for both long and short forms of isoform 1 [63]. While successful in protection of RGCs, there was limited recovery in optic nerve signal transduction. More recent work using mice with chemically-induced optic neuropathy showed that AAV delivery of either isoform 1 or 7 of *OPA1* was individually capable of rescuing mitochondrial bioenergetics in vitro and protecting functional visual parameters in vivo [64]. Together, these early experiments suggest that AAV-mediated *OPA1* gene therapy may have a role in preserving visual function, but more work is required to understand the molecular pathophysiology of ADOA.

### Gene editing

Gene editing using CRISPR-Cas9 technology could be used to correct pathogenic variants in nuclear-encoded genes. Using patient-derived induced pluripotent stem cells (iPSCs), an *OPA1* c.1334 G > A variant was corrected using CRISPR-Cas9 gene editing, improving mitochondrial structure and function [65]. Whilst it is not currently feasible to create individual gene editing strategies individually for the >500 pathogenic *OPA1* variants known to cause ADOA, this technique could be helpful for drug screening and the development of optic nerve regeneration strategies [66].

Despite the difficulty of traversing the mitochondrial double membrane, precise correction of mtDNA LHON variants using CRISPR-Cas9 has been achieved with modified guide RNA and Cas9 enzymes to localise to mitochondria [67–69]. In addition, novel efficient mitochondrial base-editing systems that do not use CRISPR-Cas9 are under active development and have shown promising results in vitro [70–73].

### Heteroplasmic shift

A disease-causing mtDNA variant can be present either in the homoplasmic state (100%) or at varying levels of heteroplasmy, in combination with the wild-type mtDNA variant. Heteroplasmy shifting is being considered for primary mitochondrial diseases to decrease the level of the pathogenic mtDNA variant in relation to the wild-type variant. In heteroplasmic individuals, clinical severity often depends on the proportion of pathogenic to wild-type mtDNA and the tissue-specific threshold required to cause biochemical impairment [74]. Selective destruction of pathogenic mtDNA can enable wild-type mtDNA to replicate more effectively, increasing its relative abundance and potentially restoring mitochondrial function. Two systems have accomplished this in mouse models using mitochondrially-targeted specific endonucleases: mitoZFN [75, 76] and mitoTALEN [77, 78]. This therapeutic strategy could be useful for LHON, but one needs to keep in mind that ~90% of LHON carriers are homoplasmic for the pathogenic mtDNA variant [79]. RGCs will also need to be targeted specifically and efficiently to be effective. Alternatively, heteroplasmy shifting could also be used in the oocytes of a woman carrying a heteroplasmic LHON mtDNA variant to decrease the level to below the threshold thought to cause disease [80].

Taking this further, mitochondrial replacement therapy (MRT) offers a potential alternative strategy to reduce the risk of transmission of pathogenic mtDNA variants from mother to child. In the UK, careful preclinical testing [81] led to the development of a novel National Health Service (NHS) clinical pathway that offered MRT to women with confirmed pathogenic mtDNA variants at homoplasmic or high heteroplasmic levels [82, 83]. This pioneering work used the pronuclear transfer technique, and this has led to the birth of eight healthy babies with heteroplasmy levels ranging from undetectable to 16% [84, 85]. Long-term monitoring of the health and heteroplasmy levels of these offspring are underway. The scientific and ethical considerations of MRT have been comprehensively reviewed elsewhere [86].

## Gene expression modification

The complex genetic basis of ADOA is a substantial barrier to the development of gene therapy. Consequently, alternative strategies that modulate gene expression at the transcriptional level have emerged as promising avenues. Most *OPA1* pathogenic variants result in premature termination of translation and reduced OPA1 protein levels, making haploinsufficiency the primary disease mechanism [15]. A therapeutic strategy is, therefore, to enhance the expression of the wild-type allele to restore normal protein levels. One way this can be achieved is with antisense oligonucleotides (ASO). As part of normal alternative splicing, some *OPA1* pre-mRNAs include an exon that signals the nonsense-mediated decay (NMD) of the transcript. NMD is a conserved pathway for quality control and gene expression regulation in normal cells [87]. A synthetic ASO has been developed to prevent the inclusion of this exon in the *OPA1* pre-mRNA, thereby upregulating wild-type *OPA1* translation in a variant-agnostic manner (Fig. 1) [88, 89]. This resulted in increased OPA1 protein production and improved mitochondrial bioenergetics in three ADOA patient-derived cell lines [88]. Intravitreal injection of this ASO in wild-type animal models was found to be safe, well-tolerated, and resulted in a dose-dependent reduction of NMD as well as a concomitant increase in OPA1 protein within RGCs. A first-in-human phase I safety trial is being planned (EUCT 2023-506290-35-00) [90, 91].

Another ASO under investigation consists of an antisense oligomer bound to a cell-penetrating peptide to enhance cell entry [92]. This molecule suppresses inhibitory elements in the regulatory region of *OPA1* mRNA, thereby enhancing wild-type protein expression. Preclinical studies demonstrated that this ASO increased OPA1 protein levels and improved mitochondrial function in patient-derived cell lines. Intravitreal injections in non-human primates confirmed retinal delivery and elevated OPA1 protein levels in RGCs [92]. A first-in-human phase 1 safety trial for this ASO is currently recruiting (NCT06461286).

While augmenting wild-type *OPA1* expression may benefit patients with null alleles, it may not address missense variants that exert dominant-negative effects by interfering with normal protein function [58]. An alternative approach is the transcriptional suppression of defective mRNA using RNA trans-splicing (Fig. 1) [93]. This technique replaces defective exons in pre-mRNA by splicing in exogenously delivered wild-type exons, producing a chimeric mature mRNA without pathogenic domains [94]. This approach has been applied to correct variants within and downstream of the GTPase domain of *OPA1*, preserving isoform expression since alternative splicing occurs upstream [95]. Further work is ongoing in different *OPA1* disease models to determine the effectiveness of such a strategy for both haploinsufficiency and dominant-negative *OPA1* variants.

In summary, gene expression modification using ASOs or RNA trans-splicing offers promising, variant-agnostic treatment approaches for ADOA. However, since these therapies target transient RNAs, they are subject to molecular turnover and require regular intravitreal injections that are likely to be needed from a young age. This poses significant burdens on patients and healthcare systems, and the cumulative risk of complications, such as endophthalmitis and chronic uveitis, warrants careful consideration.

## Optic nerve regeneration

The therapeutic approaches that have been discussed so far aim to prevent further RGC death and halt vision loss in IONs, but they cannot restore vision for patients who have already experienced significant RGC loss. For these severe end-stage cases, regenerating the optic nerve by repopulating RGCs is being explored. This is a formidable challenge because the mammalian central nervous system has a limited innate capacity for regeneration following injury [96]. A potential solution is to generate induced pluripotent stem cells (iPSCs) from an individual, correct the underlying pathogenic genetic variant, differentiate them into functional mature cells, such as RGCs, and then transplant these cells into the retinal circuitry, where they will have to integrate and form appropriate synaptic connections (Fig. 1) [96, 97]. Whilst iPSC generation, gene editing and RGC differentiation are well established methods [65, 66], the latter steps remain to be resolved.

Transplantation of iPSC-derived RGCs into the healthy retina of rats has been achieved, and some of these cells appeared to form functional synapses with host retinal neurones, and even extend along the optic nerve and react to light [98]. However, the rate of RGC integration was less than 10%, likely due to structural and immunological barriers, and few grew axons past the lamina cribrosa. Understanding and manipulating axonogenesis could prove pivotal in overcoming these physical barriers. Experiments have trialled providing neurotrophic support for RGCs to revert to an axogenic state [99, 100], and using chemoattractants [101], electrical fields [102], or physical scaffolds [103] to guide axon growth. Finally, regenerated axons must establish functional retinotopic connections with the brain. Mice optic tracts were lesioned proximal to the superior colliculus and the upregulation of neurotrophic factors increased the reformation of functional synapses [104]. However, functional visual restoration was limited. To achieve better restoration, high-contrast visual stimulation is being considered as these light-driven responses could potentially guide the organisation of RGC synapses [105].

An open label, non-randomised, patient-funded study evaluated the transplantation of autologous bone marrow-derived mesenchymal stem cells in a small number of affected individuals with LHON and *OPA1*-ADOA [106, 107] (NCT01920867 and NCT03011541). While improvements in visual acuity were noted in several eyes, concerns have been raised about the study design, including the lack of placebo and long-term follow up. It is important for clinicians and researchers to educate the patient community that optic nerve regeneration is not yet ready for clinical translation. Recognising the importance of collaboration in solving these challenging problems, an international consortium (RReSTORe) was established in 2021 to advance global scientific efforts in this area of active multidisciplinary research [96, 108].

## Future directions

Advanced therapies hold great promise for protecting and restoring vision for individuals with IONs. A major theme across all approaches is the challenge of determining whom to target and when during the disease course [109]. Studies investigating idebenone treatment for LHON demonstrated that sustained treatment provides the best neuroprotective effect. Notably, treatment responses were influenced by age, disease phase and the causative pathogenic variant [21–23, 25, 110]. Gene therapy for LHON based on allotopic gene expression is showing promise with individuals treated within one year of disease onset achieving a better visual outcome compared to what is known about the natural history of this mitochondrial genetic disorder [48, 49]. However, there are several unanswered questions, such as the potential benefit for patients who are in more chronic stages of the disease, and why a contralateral effect is seen with a unilateral intravitreal injection of the gene therapy vector. For patients with significant RGC loss, optic nerve regeneration might be the only way to restore vision. Whilst stem cell-based strategies are under preclinical investigation, the structural and functional integration of transplanted cells presents a substantial barrier to optic nerve regeneration. These challenges will need to be addressed in addition to the safety considerations. Natural history studies involving deep phenotyping and multi-omics correlations will be crucial to understanding clinical heterogeneity and differences in treatment responses [18]. Moreover, improved prognostic models will aid in stratifying patients for clinical trials. For example, biomarkers such as early optical coherence tomography (OCT) changes may identify patients with high “risk to conversion”, enabling earlier or even prophylactic treatment [111–114].

## Conclusion

The past decade has provided us with the genomic and technological tools needed to achieve breakthroughs in new therapeutics for IONs, with LHON and *OPA1*-ADOA leading the way. In addition to traditional gene replacement therapy, other exciting approaches are being actively investigated ranging from gene editing to modulation of mRNA expression, and stem cell-based optic nerve regeneration. While these advanced molecular strategies offer hope for future disease modification, it is important to recognise that visual rehabilitation, including the use of modern assistive technologies, remains essential to current care [115]. We need to maintain the momentum, with a continuing drive for innovation and collaboration being our best hope to transform the management of IONs and improve the visual outcome for affected individuals.
